# Recent Advances in Gut Microbiota in Psoriatic Arthritis

**DOI:** 10.3390/nu17081323

**Published:** 2025-04-11

**Authors:** Maria Grazia Bonomo, Salvatore D’Angelo, Valentina Picerno, Antonio Carriero, Giovanni Salzano

**Affiliations:** 1 Department of Health Sciences, University of Basilicata, Viale dell’ Ateneo Lucano 10, 85100 Potenza, Italy; salvatore.dangelo@unibas.it (S.D.); giovanni.salzano@unibas.it (G.S.); 2 Rheumatology Department of Lucania, San Carlo Hospital of Potenza, Via Potito Petrone, 85100 Potenza, Italy; valepicerno@tiscali.it (V.P.); carrieroantonio@hotmail.it (A.C.)

**Keywords:** gut microbiota, psoriatic arthritis, gut-skin-joint axis, dysbiosis

## Abstract

Psoriatic arthritis (PsA) is a chronic inflammatory disease characterized by joint inflammation and skin lesions. Recent research has underscored the critical role of gut microbiota—comprising bacteria, fungi, viruses, and archaea—in the pathogenesis and progression of PsA. This narrative review synthesizes the latest findings on the influence of gut microbiota on PsA, focusing on mechanisms such as immune modulation, microbial dysbiosis, the gut-joint axis, and its impact on treatment. Advances in high-throughput sequencing and metagenomics have revealed distinct microbial profiles associated with PsA. Studies show that individuals with PsA have a unique gut microbiota composition, differing significantly from healthy controls. Alterations in the abundance of specific bacterial taxa, including a decrease in beneficial bacteria and an increase in potentially pathogenic microbes, contribute to systemic inflammation by affecting the intestinal barrier and promoting immune responses. This review explores the impact of various factors on gut microbiota composition, including age, hygiene, comorbidities, and medication use. Additionally, it highlights the role of diet, probiotics, and fecal microbiota transplantation as promising strategies to modulate gut microbiota and alleviate PsA symptoms. The gut-skin-joint axis concept illustrates how gut microbiota influences not only gastrointestinal health but also skin and joint inflammation. Understanding the complex interplay between gut microbiota and PsA could lead to novel, microbiome-based therapeutic approaches. These insights offer hope for improved patient outcomes through targeted manipulation of the gut microbiota, enhancing both diagnosis and treatment strategies for PsA.

## 1. Psoriatic Arthritis

Spondyloarthritis (SpA) refers to a group of inflammatory diseases affecting the joints and spine, with various clinical manifestations, including peripheral arthritis, enthesitis, dactylitis, sacroiliitis, and extra-musculoskeletal involvement, such as psoriasis, uveitis, and chronic inflammatory bowel disease (IBD) [1].

The SpA family includes Psoriatic Arthritis (PsA), ankylosing spondylitis, reactive arthritis, arthritis associated with IBD, undifferentiated SpA, and juvenile-onset SpA [1].

PsA is a chronic, immune-mediated inflammatory joint disease frequently observed in combination with psoriasis (PsO), a chronic inflammatory skin condition affecting 1–3% of the global population, that typically precedes the onset of arthritis, with around 30% of PsO patients developing PsA. However, 20% of patients develop PsA before psoriasis, and in 15% of cases, both skin and joint manifestations appear simultaneously [2].

The overall prevalence of PsA ranges from 0.01% in the Middle East to 0.19% in Europe [3] and men and women are affected equally; however, the condition is less common among Asians and Black individuals and is almost absent in Native South American and Australian populations [4].

The clinical course of PsA is highly variable and the long-term adverse outcomes are often linked to delayed diagnosis, whereas early diagnosis is critical to initiating timely and appropriate treatment, preventing joint damage and deformities, reducing comorbidities, and achieving better overall outcomes.

### 1.1. Clinical Manifestations

Diagnosing PsA is challenging due to its multiple clinical manifestations, often non-specific symptoms, and the lack of a definitive biomarker. About 15% of psoriasis patients seen in dermatology clinics have undiagnosed PsA [2]. Screening psoriasis patients for PsA is thus a key step toward early disease identification.

PsA includes heterogeneous musculoskeletal and extra-musculoskeletal manifestations.

Musculoskeletal involvement is mainly classified into four types:

- Peripheral Arthritis: characterized by peripheral joint inflammation with synovitis typically presenting as asymmetric oligoarthritis before progressing to a polyarticular form [5];
- Dactylitis: characterized by uniform swelling of an entire digit due to inflammation of joints, soft tissues, and tendon sheaths [6];
- Enthesitis: characterized by inflammation of entheses i.e., sites where ligaments or tendons attach to bone [7];
- Axial involvement: symptoms include spinal pain and stiffness that improve with movement, present in up to 50% of PsA patients [8].

PsA extra-musculoskeletal features include soriasis, with plaque psoriasis being the most common phenotype, often accompanied by nail involvement; uveitis, an inflammation of the anterior segment of the eye; IBD, including Crohn’s disease and ulcerative colitis; metabolic syndrome, characterized by insulin resistance, obesity, dyslipidemia, and hypertension; cardiovascular diseases, such as myocardial infarction and stroke; and psychological disorders, such as anxiety and depression.

### 1.2. Diagnosis

To minimize delays in PsA diagnosis, dermatologists play a key role in assessing psoriasis patients for PsA signs and symptoms during every clinical visit. Unlike many other rheumatologic disorders, such as rheumatoid arthritis, PsA lacks a fully validated serum autoantibody or biomarker to facilitate early screening and diagnosis. The absence of a biomarker has been recognized as an unmet need in PsA management. Ongoing research is focused on identifying potential diagnostic and prognostic biomarkers for PsA. Due to its heterogeneous presentation, PsA can be both overdiagnosed and underdiagnosed. A diagnosis of PsA should be considered whenever a patient with psoriasis or a family history of psoriasis presents with peripheral arthritis—especially if oligoarticular or involving the distal interphalangeal joints—enthesitis, or dactylitis. Magnetic resonance imaging and ultrasonography are valuable tools for early diagnosis, particularly in cases of isolated enthesitis or inflammatory spinal pain. To minimize delays in PsA diagnosis, dermatologists play a key role in assessing psoriasis patients for PsA signs and symptoms during every clinical visit. Unlike many other rheumatologic disorders, such as rheumatoid arthritis, PsA lacks a fully validated serum autoantibody or biomarker to facilitate early screening and diagnosis. The absence of a biomarker has been recognized as an unmet need in PsA management [7,8].

### 1.3. Risks Factors and Pathogenesis

Most patients develop PsA after several years of psoriasis; therefore, identifying risk factors is crucial for early diagnosis, which can provide an opportunity to improve prognosis by enhancing long-term treatment outcomes for patients. Knowledge of risk factors comes from retrospective studies, as the onset of PsA in psoriasis patients may take decades. Several risk factors for PsA development have been identified and classified as modifiable and non-modifiable (Table 1).

Solmaz et al. (2018) [9] found that a family history of psoriasis is associated with an increased risk of PsA and impacts skin disease phenotypes and PsA severity. The presence of a family history of PsA has been proposed for screening among psoriasis patients. Thus, PsA tends to occur more frequently in individuals with a family history of psoriasis, PsA, or seronegative spondyloarthritis [10]. Multiple genes involved in PsA have been identified. Significant polymorphisms are found within inflammatory pathways such as IL-17/IL-23, NFkB signaling pathways, and additional pathways like IL-23R, tumor necrosis factor alpha-induced protein 3 (TNFAIP3), IL-12B, NOS2, TNFα-238 A/G, and TNFα-857 T/C. These are classified as non-HLA loci. The effect of these polymorphisms is minor, limiting their usefulness in predicting PsA among psoriasis patients. The only region where specific genetic markers for PsA have been identified is the human leukocyte antigen (HLA) locus, particularly HLA-B and HLA-C [9,11].

Psoriasis-related factors include the age of psoriasis onset, localization, severity, and nail involvement. In the first case, there is conflicting evidence correlating psoriasis onset age with the risk of developing PsA [12]. In the second case, scalp involvement and intergluteal fissure psoriasis may be associated with an increased risk of PsA development. The relationship between psoriasis severity and PsA is well supported. Psoriatic nail involvement is linked to a higher risk of PsA development, as it indicates an abnormal inflammatory process simultaneously affecting multiple anatomical structures [12].

Environmental factors such as physical and emotional trauma, infections, and vaccinations are believed to play a predominant role in PsA development in genetically predisposed individuals. Heavy lifting, injuries, infections requiring antibiotics, and vaccinations have been associated with disease onset [9].

Various metabolic abnormalities, including obesity, hyperlipidemia, and hyperuricemia, have been linked to an increased risk of developing PsA. Patients with the disease often suffer from diabetes mellitus or hyperinsulinemia and hypertension. They are frequently obese, and PsA incidence rises in correlation with body mass index. Visceral fat can simultaneously contribute to metabolic disorders and systemic inflammation. In fact, visceral adipose tissue can synthesize pro-inflammatory mediators, known as adipokines, when stimulated by pro-inflammatory cytokines. Obese individuals show increased serum leptin concentrations (which have a pro-inflammatory role) and decreased serum adiponectin concentrations (which, conversely, exhibit anti-inflammatory properties). Elevated leptin levels may contribute to endothelial dysfunction, oxidative stress, thrombocytosis, myointimal thickening, and, ultimately, atherosclerosis. Additionally, leptin may activate osteoclasts, thus participating in erosive bone damage in PsA [10].

The role of smoking in PsA pathogenesis is not entirely clear: an inverse association between smoking and disease development has been reported, suggesting that non-smokers are at higher risk. Eder et al. [13] described the paradox of smoking only in HLA-C*06-negative cases. The biological explanation for smoking’s protective effect has been hypothesized through reduced IL-1b and IL-8 expression and an altered Toll-like receptor pathway response to harmful agents.

Long-term use of paracetamol and nonsteroidal anti-inflammatory drugs (NSAIDs) may be associated with an increased risk of PsA. Establishing a causal link between medications and disease development is challenging, as PsA patients may experience a preclinical phase before formal diagnosis, during which they might have had joint pain without objective signs of inflammation and could have used painkillers [9].

PsA pathogenesis is based on a multifactorial model: it involves the interaction of genetic predisposition, environmental triggers such as biomechanical stress, and local factors depending on the disease site (joints, skin, spine/entheses), along with innate and adaptive immune responses that combine to influence observed clinical phenotypes. Based on these concepts, the term “psoriatic disease” can be used to emphasize that inflammation in patients is systemic and not necessarily limited to the joints. Common inflammatory and metabolic pathways may be activated across different tissues and cells, including endothelial cells, adipose tissue, synovium, and skin. Innate immune system cells in the skin or entheses, activated by environmental or mechanical stimuli within an appropriate genetic context, drive the expansion of type 1 cells [CD4+ T helper 1 (TH1) or CD8+ cytotoxic T cells (Tc1)] or type 17 cells [CD4+ T helper 17 (TH17) or CD8+ cytotoxic T cells (Tc17)] [11].

#### 1.3.1. Biomarkers

A biological marker is any component identified through genomics, proteomics, or transcriptomics approaches associated with the pathophysiology, clinical course, or outcome of a specific disease. The availability of a biomarker facilitates the identification of individuals who may develop PsA [14]. Genomic biomarkers include HLA alleles: HLA-B*27, -B*38, -B*39, and -B*8 have been identified as specific markers for PsA among psoriasis patients. The HLA-B*27 allele has been associated with early development of PsA among psoriasis patients, along with polymorphic alleles of the gene encoding beta-2-microglobulin, which may modulate the composition of the gut microbiota, currently being investigated as a key player in the pathogenic scenario of spondyloarthritis [10]. HLA-C*06 has been associated with a delayed onset of PsA. The HLA-B*27:05:02 allele is positively associated with enthesitis, dactylitis, and symmetric sacroiliitis, while the HLA-B*08:01:01 and HLA-C*07:01:01 haplotype is associated with joint fusion and deformities, asymmetric sacroiliitis, and dactylitis [11,14].

Regarding clinical markers, severe psoriasis, defined by body surface area (BSA) or the number of involved sites, is a clinical characteristic associated with an increased risk of PsA. Greater skin psoriasis extent is believed to be correlated with a higher systemic inflammatory burden, which in turn may trigger joint inflammation [15]. The markers hs-CRP (high-sensitivity C-reactive protein), OPG (osteoprotegerin), and MMP-3 (Matrix Metalloproteinase-3) have been useful in distinguishing PsA patients from those with psoriasis without arthritis. In recent years, the C-X-C motif chemokine 10 (CXCL10) has emerged as a biomarker for PsA development in psoriasis patients. Individuals who later developed PsA had higher serum levels of CXCL10 compared to those who did not. Serum levels of this biomarker decreased after disease onset. Among cellular biomarkers, osteoclast precursor (OCP) was found in one-third of psoriasis-only patients and in most PsA patients. Patients also developed an antibody against a dendritic cell-specific transmembrane protein (DC-STAMP), which was associated with OCPs and could serve as an additional biomarker for early PsA identification [14].

#### 1.3.2. Innate Immune System

Innate immune system cells are involved in disease pathogenesis. Dendritic cells activate the adaptive immune response by presenting antigens and secreting cytokines to generate distinct subsets of T cells. In PsA synovial fluid, dendritic cells predominate and remain sensitive to Toll-like receptor ligands. Dendritic cells show upregulated expression of Toll-like receptor 2 but not Toll-like receptor 4, inducing a T helper 1 (TH1) cellular response with increased production of TNFα, interferon-γ, and IL-12. The prevalence of synovial CD8+ T cells in PsA may partly be explained by inappropriate dendritic cell activation [16].

Activated macrophages promote various pro-inflammatory mechanisms in the synovium. Classically activated M1 macrophages are pro-inflammatory and play a central role in host defense against infections, while alternatively activated M2 macrophages are associated with anti-inflammatory responses and tissue remodeling. The abundant secretion of pro-inflammatory cytokines by macrophages is a hallmark of PsA. Macrophages also secrete large amounts of matrix metalloproteinases (MMPs) and inducible nitric oxide synthase (iNOS), present antigens to T and B cells, and drive bone resorption, suggesting a predominant M1 macrophage phenotype in inflammatory arthritis [17].

A population of immune cells known as MAIT (Mucosal-associated invariant T) cells and NK (Natural Killer) cells are implicated in autoimmune diseases. MAIT cells are enriched in the synovial fluid of ankylosing spondylitis patients and produce IL-17A. Additionally, increased IL-17 production by CD8+ MAIT cells has been demonstrated in psoriatic skin and synovial fluid in PsA [18]. Thus, MAIT cells serve as an alternative source of IL-17A at inflammation sites.

Natural killer cells play both protective and pathogenic roles, regulated by activating and inhibitory receptors. Synovial natural killer cells in PsA show increased expression of activation markers CD69 and NK-p44, with greater production of INF-γ and TNF-α compared to peripheral blood cells. Mast cells expressing IL-17A are present in synovial fluid and synovial membrane. Histamine and tryptase released by mast cells can promote synovial angiogenesis and neutrophil recruitment, suggesting that mast cells may play an active role in the inflammatory cascade [19].

#### 1.3.3. Adaptive Immune System

Psoriatic arthritis (PsA) is a multifactorial disease driven by both genetic and environmental factors. However, the relative contribution of these components to disease pathogenesis remains poorly understood. A defining feature of PsA is inflammation of the synovial membrane, a specialized connective tissue lining the joint capsule and articulating surfaces of bones. This inflammation is driven by immune system activation and associated pro-inflammatory cytokine expression, including increased levels of Tumor Necrosis Factor-alpha (TNF-α), which primarily occur in joints, synovium, and skin [20]. Table 2 outlines the key cytokines involved in PsA pathogenesis.

The IL-23/Th-17 axis is a well-established driver of PsA onset and progression. IL-23 is a heterodimeric cytokine secreted by innate immune cells, such as dendritic cells and macrophages. Its receptor, IL-23R, is expressed on Th17 cells, gamma-delta T cells, and other lymphoid cells. Activation of IL-23R leads to the phosphorylation of Jak2 and Tyk2 kinases, which activate transcription factors STAT3 and ROR-γ, promoting Th17 survival and expansion. Th17 cells then secrete IL-17, a major contributor to PsA pathogenesis [16]. IL-17 binds its receptor (IL-17R), present in various tissues, triggering Nuclear Factor-kβ (NF-kβ) activation. This pathway promotes MMP expression in synovial fluid and cartilage, leading to collagen degradation and cartilage erosion [21].

Key players in the immune cascade include CD8+ T lymphocytes, CD4+ TH1, TH17, and TH22 lymphocytes. CD8+ T lymphocytes are activated following antigen presentation via MHC-I by dendritic cells. TH1 lymphocytes express cytokines such as INF-γ and IL-12, contributing to skin inflammation by activating keratinocytes. TGF-β, IL-6, and IL-1β promote differentiation towards TH17, leading to IL-17 secretion (mainly the A isoform) and the release of other cytokines such as IL-21, IL-9, and IL-23. Once activated, TH17 cells can also produce IL-21, IL-22, and CCL20 chemokine. IL-17A activates macrophages, keratinocytes, epithelial cells, dendritic cells, endothelial cells, fibroblasts, chondrocytes, neutrophils, osteoclasts, and osteoblasts, which in turn release pro-inflammatory mediators, including IL-1, IL-6, IL-8, TNF-α, MMP-9, GM-CSF, iNOS, and RANK, amplifying the inflammatory cascade [10].

TH22 lymphocytes develop from naive TH cells upon stimulation by TNF-α and IL-6 and synthesize IL-22, stimulating keratinocyte activation and promoting immune responses. Treg lymphocytes suppress effector T cell activation and proliferation, playing a crucial role in immune regulation and tolerance towards self-antigens. In psoriasis, alterations in natural Treg lymphocytes appear to be present. The IL-23/IL-17 pathway also plays a homeostatic role in defending against fungal and bacterial pathogens at mucosal barriers, but its dysregulation facilitates local inflammation in PsA [22].

The engagement of IL-23 with IL-23R results in signaling primarily through Janus kinase 2 (JAK2) and tyrosine kinase 2 (TYK2), leading to phosphorylation of STAT3 and subsequent expression of RORγt and IL-17 production [23]. IL-23/IL-23R appears to be a central orchestrator of pathogenesis in PsA and spondyloarthritis [24,25]. Alongside immunological alterations, a key factor in psoriatic lesion development is neoangiogenesis, which facilitates leukocyte migration from peripheral blood and results in persistent immune cell infiltration in inflamed joints.

Fibroblast-like synoviocytes in PsA exhibit an abnormal phenotype characterized by increased proliferation and invasiveness, transforming the synovial membrane into a tumor-like surface capable of destroying articular cartilage and bone. Vascular changes include increased synovial blood vessels, elongation, and dilation. Immature blood vessels indicative of a plastic condition, reduced endothelial apoptosis, and increased synovial expression of angiogenic growth factors such as VEGF, VEGFR1, VEGFR2, angiopoietin 1 and 2, and their receptor TIE2 have been identified. Despite increased vascularization, psoriatic synovium remains hypoxic, with oxygen levels inversely correlated with synovitis, immune cell infiltration, and pro-inflammatory mediator activation [25].

Metabolic shifts towards glycolysis drive T cell, macrophage, dendritic cell, and synoviocyte activation, perpetuating the inflammatory response. Immune cells adapt to hypoxia by altering their metabolism, which activates key signaling pathways, cytokine production, and systemic inflammation. This metabolic-immune interplay may contribute to increased PsA-associated comorbidities, even in the absence of conventional risk factors. Lymphocytes, particularly T and B cells, are frequent immune infiltrates in PsA. Lymphoid aggregates have been observed in synovium, with their absence associated with disease remission. PsA development results from interactions between genetic predisposition, environmental triggers (e.g., biomechanical stress), local factors and innate and adaptive immune responses, influencing diverse clinical phenotypes. Genome-wide association studies, heritability studies, and HLA allele research support a strong genetic component in PsA [16]. Twin studies in European populations indicate an 80–100% higher disease likelihood in monozygotic twins versus dizygotic twins [26]. An Icelandic study reported a 40% increased PsA risk among first-degree relatives compared to unrelated controls [27]. Four pathological phenotypes linked to MHC class I alleles and haplotypes have been proposed in PsA: skin-predominant phenotype associated with the presence of HLA-B57:01 and HLA-C06:02 alleles; synovial-predominant phenotype associated with the presence of HLA-B08:01:01, HLA-C07:01:01 alleles and the HLA-B08:01:01-HLA-C07:01:01 haplotype; enthesis-predominant phenotype associated with the presence of HLA-B*27:05:02 allele; and mutilans phenotype (PAM), a severe form of PsA that is potentially linked to interactions among three genetic variants and cellular pathways [28].

Non-HLA loci associated with PsA include IL23R and RUNX1. Trauma is a known psoriasis factor, with the Koebner phenomenon causing psoriatic plaques post-injury. A longitudinal study found that 24.6% of PsA patients experienced local trauma before disease onset [29].

Enthesitis, inflammation at tendon and ligament attachment sites, is a key PsA feature. Entheses, which transmit mechanical forces between muscles and bones, are extra-articular structures subject to biomechanical stress, often affecting lower limbs [30]. Prostaglandin E2 (PGE2) is an early mediator that facilitates immune cell influx and osteitis onset while enhancing IL-17 production, linking it to PsA pathogenesis. IL-17 amplifies enthesitis by activating mesenchymal cells, promoting neutrophil recruitment via GM-CSF, IL-6, and IL-8 release. This inflammation leads to new bone formation at affected sites, such as calcaneal spurs and spinal syndesmophytes [11,31].

### 1.4. Pharmacological Treatment for Psoriatic Arthritis

The management of PsA has advanced significantly in the last decade due to early diagnosis, progress in pharmacological therapies, and the broader application of multidisciplinary approaches. Improved understanding of PsA has led to the development of new innovative drugs such as biotechnological and small molecules drugs. These drugs improve signs and symptoms of inflammation and inhibit joint damage in peripheral joints, enhancing quality of life and functional status. Consequently, it is now possible to effectively modify the disease course. Current therapeutic goals include achieving symptom remission, preventing early PsA damage, or halting PsA progression in established cases [32].

#### 1.4.1. Conventional Pharmacological Treatments

This category includes non-steroidal anti-inflammatory drugs (NSAIDs), corticosteroids, and conventional synthetic disease-modifying antirheumatic drugs (csDMARDs). csDMARDs, used primarily for peripheral involvement, include methotrexate (MTX), leflunomide (LEF), cyclosporine (CsA), and sulfasalazine (SSZ). MTX, LEF, and CsA are effective for musculoskeletal and cutaneous manifestations, while SSZ is effective only for arthritis. csDMARDs are often prescribed for mild disease due to their low cost [32].

#### 1.4.2. Biologic Drugs

Biologic disease-modifying antirheumatic drugs (bDMARDs) represent a relatively new class of drugs, with the first molecules introduced approximately 20 years ago. These immunosuppressive agents target specific pro-inflammatory cytokines involved in PsA pathogenesis, effectively inactivating the molecular target and suppressing the inflammatory process. They have profoundly changed the treatment of arthritis and autoimmune diseases in general.

The most represented class includes anti-TNF-α drugs (Infliximab, Etanercept, Adalimumab, Golimumab, Certolizumab pegol), followed by drugs targeting the IL-12/IL-23 axis (Ustekinumab, Guselkumab, Risankizumab) and IL-17 (Secukinumab, Ixekizumab, Bimekizumab). These drugs are well-tolerated and have a good safety profile. The most significant side effect is an increased risk of infections. Many molecules in this category are first-line treatments for other spondyloarthritis spectrum diseases, such as psoriasis, uveitis, and inflammatory bowel diseases, highlighting shared pathogenic mechanisms and inflammatory pathways across these conditions [32].

The above-described originator drugs, meaning the first biologic formulations developed and approved before the introduction of biosimilars, are not the only ones available for use. Biosimilar versions of Infliximab, Adalimumab, and Etanercept are currently used for PsA treatment. According to the WHO, ‘a biosimilar medicinal product is a highly similar biological medicine to another biological medicine already authorized in the EU (the so-called reference medicine)’. Biosimilar use is significant due to their lower costs compared to original biologic drugs, which represent a substantial financial burden.

#### 1.4.3. Small Molecules in the Treatment of Psoriatic Arthritis

Small molecules, such as apremilast and Janus kinase (JAK) inhibitors, represent an important therapeutic option for PsA. Apremilast, a phosphodiesterase-4 (PDE4) inhibitor, modulates inflammatory pathways by increasing intracellular cAMP levels, leading to reduced cytokine production. It is particularly useful in patients with mild to moderate disease who may not require biologic therapy. JAK inhibitors, such as tofacitinib and upadacitinib, target intracellular signaling pathways involved in immune activation, offering an effective alternative for patients with inadequate response to conventional or biologic DMARDs. These oral therapies provide additional flexibility in PsA management, particularly for patients seeking non-injectable treatment options [33].

## 2. Gut Microbiota

### 2.1. Development, Diversity and Functions

The microbiota refers to the collection of microorganisms, including bacteria, fungi, viruses, and archaea, that live inside and on the human body in perfect symbiosis. It is well established that bacterial populations differ not only between individuals but also within the same individual [33]. The human gut contains approximately 1000 different species of bacteria. To date, 50 phyla of bacteria have been described and 5/6 of which dominate in human gut microbiota [34,35].

Gut microbiota development begins before birth, with evidence of bacteria in the placenta, umbilical cord, and amniotic fluid [36,37]. Colonization of the fetus by maternal microbes is influenced by factors such as delivery mode, feeding practices, and antibiotic use [38]. The microbial composition varies along the digestive tract; the gut microbiota is dominated by seven bacterial phyla: Firmicutes (*Clostridium*, *Eubacterium*, *Ruminococcus*), Bacteroidetes (*Bacteroides*, *Prevotella*), and Actinobacteria, Fusobacteria, Proteobacteria, Verrucomicrobia, and Cyanobacteria [39,40]. Based on microbial composition, the gut microbiota can be classified into three enterotypes [41]: Enterotype 1, dominated by *Bacteroides*, involved in carbohydrate and protein metabolism; Enterotype 2, dominated by *Prevotella*, specialized in mucin degradation and vitamin synthesis; and Enterotype 3, dominated by *Ruminococcus*, responsible for mucin degradation and sugar transport.

The interaction between various microbes is essential for a productive microbiota. However, Foster and colleagues [42] report a mathematical analysis showing that cooperation between microorganisms is not always beneficial; instead, it can sometimes compromise microbiota stability. According to this study, intestinal microbiota homeostasis is maintained through positive and negative feedback mechanisms [43].

Cooperation can become destabilizing when it introduces positive feedback loops that generate “runaway effects.” For example, when two species cooperate, an increase in the abundance of one species leads to an increase in the abundance of the second, which in turn increases the abundance of the first, and so on [44,45]. If these increases are not sufficiently regulated by other constraints, uncontrolled growth of cooperative species can occur, leading to the collapse of competing populations and destabilization of the community due to compromised diversity. In this regard, the presence of species that primarily engage in competitive interactions within a microbial community is crucial. Contrary to what might be expected, such species help stabilize the community by dampening positive feedback loops or preventing cooperation from leading to collapse [46].

Foster et al. [42] proposed three negative feedback mechanisms through which the host exerts selective pressure on cooperating microbes to maintain their homeostasis in the gut: when certain species in the microbiota increase rapidly, the host induces a targeted immune response that disrupts the positive feedback between cooperative species; the host can block cooperative interactions between species by spatially separating them, thereby reducing their interactions; and the host can nourish microorganisms to reduce cooperation among species by providing alternative carbon sources so that these species no longer depend as strongly on their cooperative partners [47]. However, it is still unknown whether these stability-promoting mechanisms are intentional microbiota engineering adaptations of the host or merely fortuitous byproducts of existing host traits and adaptations [46].

The *Baas Becking hypothesis* proposed that microbes distributed in a given environment, along with the environment itself, determine microbial biodiversity [48]. For example, the diet and lifestyle of the Hadza hunter–gatherers of Tanzania have influenced the richness and diversity of their gut microbiota compared to urban farming populations in Italy and Africa [49]. The predominance of *Prevotella*, *Treponema*, and unclassified Bacteroidetes in the Hadza aids in digesting fibrous plant-based foods [50]. Studies on the microbiota functionalities in this group have shown that metabolic pathways are involved in digesting a broad spectrum of carbohydrates, degrading branched-chain amino acids, and synthesizing aromatic amino acids.

In a Western lifestyle, the intake of more amino acids, lipids, cholesterol, and dairy products supports the growth of *Faecalibacterium*, *Ruminococcus*, *Bifidobacterium*, *Bacteroides*, *Blautia*, *Bilophila*, and *Alistipes*. Conversely, the consumption of sugar and complex carbohydrates in non-industrialized ethnic societies supports the growth of *Prevotella* [49].

The microbial profile of rural and urban cohorts from seven ethnic groups across nine Chinese provinces revealed nine main bacterial groups (*Balutia*, *Clostridium*, *Ruminococcus*, *Faecalibacterium*, *Subdoligranulum*, *Roseburia*, *Coprococcus*, *Bacteroides*, and *Phascolarctobacterium*), linked to their respective ethnicities, geographies, and lifestyles [51].

In India, six main bacterial groups (*Faecalibacterium*, *Eubacterium*, *Clostridium*, *Blautia*, *Ruminococcus*, and *Roseburia*) were identified across 15 ethnicities from four geographical locations.

Exposure to extreme environments, such as high altitudes (above 1493 m), is also responsible for alterations in gut microbiota due to reduced atmospheric oxygen pressure. This explains why high-altitude visitors—such as pilgrims, hikers, mountaineers, and military personnel—often suffer from nonspecific gastrointestinal complications [52,53]. Even cold environmental stress is a factor that alters gut microbiota and energy homeostasis.

The gut microbiota performs essential roles in four domains of the human body: metabolic, structural, protective, and neurological.

#### 2.1.1. Metabolic Function

The gut microbiota is responsible for metabolizing food into bioactive components. Approximately 85% of carbohydrates, 66–95% of proteins, and all fats are digested within the gastrointestinal tract. Gut bacteria convert indigestible carbohydrates—such as cellulose, hemicellulose, starch, pectin, oligosaccharides, and lignin—into short-chain fatty acids (SCFAs) like acetate, propionate, and butyrate, which provide energy and regulate inflammation [54]. These metabolic products are synthesized primarily by Firmicutes, Bacteroidetes, and some anaerobic gut microorganisms [54]. Although dietary fibers, including lignin, non-starch polysaccharides, and oligosaccharides such as fructooligosaccharides (FOS) and galactooligosaccharides (GOS), are resistant to host digestive enzymes [55], gut bacteria can utilize them thanks to a series of enzymes classified into four glycosidase families: glycoside hydrolases (153 subfamilies), glycosyltransferases (106 subfamilies), polysaccharide lyases (28 subfamilies), and carbohydrate esterases (16 subfamilies) [56]. In the large intestine, bacteria ferment all dietary fibers, releasing gases (methane, hydrogen, and carbon dioxide), SCFAs (formate, acetate, propionate, butyrate, valerate, isovalerate, and hexanoate), small amounts of organic acids (lactate and succinate), and alcohols (methanol and ethanol).

In the colon, undigested proteins are broken down into peptides, amino acids, and metabolites by bacterial proteases and peptidases. Metabolites derived from proteolysis are classified into neuroactive compounds (nitric oxide, tryptamine, and phenylethylamine), sulfur-containing metabolites (H_2_S), aromatic compounds (phenol, p-cresol, and indole), polyamines (spermine, spermidine, and cadaverine), SCFAs (isobutyrate, 2-methylbutyrate, and isovalerate from branched-chain amino acids), and ammonia [57,58].

The classification of gut bacterial proteases and peptidases is documented in the MEROPS database [59], which identifies *Clostridium* spp., *Bacteroides* spp., *Lactobacillus* spp., and others as the bacteria with the highest diversity of proteases [60]. Dietary proteins also influence gut microbial composition: a diet rich in beef increases *Bacteroides* and *Clostridia* populations but negatively affects *Bifidobacterium adolescentis* proliferation [61]. On the other hand, legume proteins, such as those in peas, promote the growth of both *Bifidobacterium* and *Lactobacillus*, as also whey proteins, which inhibit the growth of pathogens, such as *Bacteroides fragilis* and *Clostridium perfringens* [62,63].

An interesting aspect is that proteins and amino acids, in association with commensals, influence food choices. This has been observed in *Drosophila melanogaster*, where gut bacteria *Acetobacter pomorum* and *Lactobacillus* spp. influenced the flies′ preference for sucrose-rich foods [64]. Different bacteria are responsible for producing various products after protein catabolism: GABA is produced by the abundance of *Lactobacillus* spp., *Bifidobacterium* spp., and *Lactococcus lactis*; norepinephrine by *Escherichia* spp. and *Bacillus* spp.; dopamine by *Bacillus* spp.; histamine and serotonin by *Streptococcus* spp., *Escherichia* spp., and *Enterococcus* spp. [60,65].

Regarding lipids, the focus is more on the effects of fat intake on gut bacterial populations rather than the microbiota’s role in their breakdown. High-fat diets have been shown to induce microbial dysbiosis, increase adiposity, and cause inflammation in adipose tissue.

A high-fat diet promotes an increase in *Bilophila wadsworthia*, a pathogen able to reduce sulfites and induce a pro-inflammatory Th1 cell-mediated response [66]. Saturated fat-rich diets increase the population of *Bacteroides*, *Bilophila*, and *Faecalibacterium prausnitzii*, whereas unsaturated fatty acids increase lactic acid bacteria, *Bifidobacteria*, and *Akkermansia muciniphila* [67,68].

Special attention is also given to polyphenols, which are inactive (e.g., flavones, flavonols, flavanones, flavanols, catechins, anthocyanins, isoflavones, hydroxycinnamic acids, lignans) and are transformed into active compounds after sugar removal by the microbiota in the small gut [69]. Polyphenols, such as hydroxycinnamic acids, are commonly esterified to sugars, organic acids, and lipids, which microbial esterases transform into aglycones. Other dietary polyphenols, such as ellagic acid and ellagitannins, are transformed into anti-inflammatory metabolites like urolithin A by *Gordonibacter* spp. [70]. Polyphenols can also inhibit the growth of pathogenic bacteria such as *Clostridium perfringens*, *Clostridium difficile*, and *Bacteroides* spp. [58].

#### 2.1.2. Structural Function

The microbiota plays a critical role in the development and maintenance of the intestinal barrier, through its influence on the production of mucus, tight junction proteins, and the epithelial cell cycle.

Mucus is produced by goblet cells, forming a protective layer against physical, chemical, and biological damage. This layer is composed of gel-forming mucins, primarily MUC2 in the large intestine, which act as the first barrier to bacterial colonization [71]. Some gut bacteria, such as *Akkermansia muciniphila* and *Bacteroides thetaiotaomicron*, utilize mucus as an energy source by degrading mucins with glycosidases and proteases [72]. The integrity of the epithelial barrier is ensured by the interaction of tight junction proteins, such as occludins, claudins, and zonula occludens, which connect epithelial cells. Studies suggest that *A. muciniphila* and *Faecalibacterium prausnitzii* enhance intestinal permeability by increasing the expression of tight junction proteins [73]. Furthermore, the microbiota influences the epithelial cell cycle, stimulating proliferation, differentiation, and apoptosis. This function is mediated by metabolites like SCFAs, which regulate the proliferation and differentiation of epithelial cells [74].

#### 2.1.3. Protective Function

The intestinal microbiota serves as a shield against pathogenic microorganisms by limiting their colonization through mechanisms such as competition for nutrients and adhesion sites, production of antimicrobial substances, and modulation of host immune responses. Commensal bacteria compete with pathogens for nutrients and ecological niches. For example, *Lactobacillus* spp. produces lactic acid, lowering the intestinal pH and inhibiting the growth of pH-sensitive pathogens like *Salmonella typhimurium* [75]. The microbiota also produces bacteriocins, small antimicrobial peptides, and SCFAs like butyrate, which exert bactericidal or bacteriostatic effects [76]. Moreover, the microbiota modulates innate and adaptive immune responses. For instance, it induces the production of secretory IgA, which neutralizes pathogens and toxins [77]. Certain bacteria, like *Bacteroides fragilis*, stimulate regulatory T cells (Tregs), maintaining immune tolerance and preventing inflammatory diseases [78].

#### 2.1.4. Neurological Function

The gut–brain axis highlights the bidirectional communication between the gut microbiota and the central nervous system (CNS). This interaction occurs through neural, endocrine, and immune pathways, as well as microbial metabolites. The vagus nerve is a major conduit for gut–brain communication. It transmits signals from the gut to the brain, influencing mood, behavior, and stress responses. For instance, *Lactobacillus rhamnosus* was shown to affect GABA receptor expression and reduce anxiety-like behavior in mice through vagal pathways [79]. The microbiota also influences the endocrine system by producing neuroactive compounds, such as serotonin, dopamine, and gamma-aminobutyric acid (GABA). These neurotransmitters can affect CNS functions. For example, 90% of the body’s serotonin is synthesized in the gut by enterochromaffin cells under the influence of microbiota-derived metabolites like SCFAs [80]. Furthermore, the gut microbiota modulates immune responses, indirectly affecting brain function. Pro-inflammatory cytokines, such as interleukin-6 and tumor necrosis factor-alpha, can cross the blood–brain barrier and influence neurological processes [81]. Lastly, microbial metabolites, such as SCFAs, phenolic compounds, and indole derivatives, impact brain function by crossing the blood–brain barrier or acting on peripheral receptors. For example, butyrate has anti-inflammatory and neuroprotective properties, reducing microglial activation and promoting neuronal survival [82].

The composition of the microbiota can vary significantly depending on body site, age, diet, lifestyle, comorbidities, hygiene levels, medication use and environmental conditions and maintaining microbial balance is crucial for preventing disease and promoting overall well-being [83]. Different anatomical sites host distinct microbial communities, each playing a crucial role in modulating host physiology [84]. The resident microbial community in sites such as the skin and gastrointestinal tract has a significant impact on disease susceptibility, influenced by both genetic and environmental factors [85]. Over the last few years, the influence of the microbiota has drawn increasing scientific attention.

### 2.2. Microbiota and Disease

Research on the microbiota has greatly improved our understanding of microbial species composition across different body parts, as well as the changes that occur in various diseases [83]. The microbiota is increasingly being studied in the etiology of immune-mediated inflammatory diseases.

The skin microbiota is examined to assess the host-microbe symbiotic relationship and to understand its contribution to skin diseases. Since PsA develops in individuals with psoriasis, studying differences in skin microbiota composition between those who develop PsA and those who do not could provide unique insights into disease pathogenesis. Such information may also help in identifying individuals predisposed to developing arthritis within the psoriatic population [85].

The skin maintains a healthy microbial ecosystem by producing antimicrobial proteins and peptides. This homeostasis depends heavily on the stratum corneum, the outermost layer of the epidermis, composed of 15 layers of keratinized, anucleate corneocytes. These cells originate from stem cells in the basal layer through the keratinization process [86].

Disruptions in the skin microbiota have been implicated in psoriasis pathogenesis, and since up to 30% of psoriasis patients develop PsA, alterations in the skin microbiome are also thought to contribute to PsA development [87].

Beyond the skin, the gut microbiota plays a pivotal role in immune regulation. The human gut microbiota contains approximately 3.3 million protein-coding genes (100 times more than the human genome). The NIH project (Human Microbiome Project, HMP) was launched to better understand and define this human microbiota [88].

The human gut microbiota is determined by genetic, epigenetic, and dietary factors. As diets and overall health change, so does the composition of the human gut microbiota over time. Interestingly, disturbances in gut flora can be associated with various forms of inflammation and autoimmune diseases. There is a correlation between the gut and joints; in fact, the concept of the “skin-joint-gut axis”, which has been applied to PsA, refers to the relationship between the gut microbiota, the skin, and joint function [89].

### 2.3. Gut Dysbiosis

The proper functioning of the gut microbiota relies on a stable cellular composition, primarily consisting of bacteria belonging to the phyla Bacteroidetes, Firmicutes, and Actinobacteria, with a smaller proportion of Proteobacteria [41]. Significant changes in the ratio of these bacterial populations or the expansion of new bacterial groups lead to an imbalance, known as dysbiosis, which can often have pathological consequences [90].

Dysbiosis has been associated with a wide range of diseases, sometimes contributing to their development or exacerbating their severity. It is particularly characteristic of chronic IBD, such as ulcerative colitis and Crohn’s disease [91], as well as metabolic disorders [92], autoimmune diseases and neurological disorders [93].

#### Factors Contributing to Dysbiosis and Consequences

Several endogenous and exogenous factors influence the microbial composition of the gut, with effects ranging from transient and harmless to long-lasting and detrimental. It is important to consider that a single factor is not sufficient to induce dysbiosis, as the gut microbiota is highly adaptable to changes in nutrient availability and environmental conditions. However, the combined actions of several factors can alter the composition of the microbiota until a “critical point” is reached, which then leads to pathological states [94]. Several factors contribute to the alteration of gut microflora composition, including diet, various medications, the immune system, and the microbiota itself. The threshold required to trigger dysbiosis largely depends on the bacterial groups involved. Large variations in the Bacteroidetes and Firmicutes phyla may remain without pathological consequences, while an increase in the quantities of minor groups can cause disruption. This is the case with Enterobacteriaceae, for example, which normally represent a small fraction of the gut microbiota but can become problematic if they expand excessively [95]. Thus, there are “key microorganisms” that can have beneficial or harmful effects depending on their abundance, as listed in Table 3.

The human body hosts a variety of microorganisms that play a crucial role in maintaining health and influencing disease. Among these, several bacterial genera have been identified as either beneficial or potentially harmful, depending on their abundance and interactions with the host.

One of the beneficial bacteria is *Akkermansia muciniphila*, a Gram-negative obligate anaerobe known for its anti-inflammatory effects [96]. Studies suggest that its presence is reduced in individuals suffering from IBD, obesity, and type 2 diabetes (T2D), but it increases after metformin treatment and in fish-oil-fed mice [97]. Another important genus, *Bacteroides* spp., plays a role in activating CD4+ T cells. Its levels increase with an animal-based diet and obesity, and *Bacteroides vulgatus* has been positively correlated with insulin resistance [90].

In contrast, *Bifidobacterium* spp., a Gram-positive obligate anaerobe, contributes to gut health by producing SCFAs, improving the gut mucosal barrier, and lowering intestinal lipopolysaccharide (LPS) levels [98]. Its abundance decreases in obesity and in smokers but is elevated in individuals with Rett syndrome. It is often used as a probiotic due to its beneficial properties [99]. Some bacteria, however, are associated with pro-inflammatory effects. *Bilophila* spp., a Gram-negative obligate anaerobe, is known to promote inflammation, with increased levels observed in colitis and lard-fed mice, while its presence is reduced in individuals with autism [96]. Similarly, *Clostridium* spp., another Gram-positive obligate anaerobe, is involved in the generation of TH17 cells. Although some species within this genus cause botulism and tetanus, others have been linked to metabolic disorders such as obesity, T2D, and Rett syndrome [97].

The microbiome also includes bacteria with potential pathogenic effects. *Dialister* spp., a Gram-positive obligate anaerobe, is commonly found in individuals with obesity and periodontitis but is reduced in those with autism [96]. *Enterobacter* spp., a Gram-negative facultative anaerobe, includes pathogenic species, with *Enterobacter cloacae* being capable of inducing obesity in germ-free mice [90]. Additionally, *Escherichia coli*, a well-known Gram-negative facultative anaerobe, is often increased in individuals with IBD and T2D due to its role in Toll-like receptor (TLR) activation [98].

Several bacterial genera contribute to gut homeostasis. *Eubacterium* spp. produces SCFAs and phenolic acids, but its abundance decreases in IBD, atherosclerosis, and T2D [99]. *Faecalibacterium prausnitzii* is another Gram-positive obligate anaerobe known for its SCFA production and anti-inflammatory effects. Its reduced levels are associated with IBD, obesity, and metabolic disorders [96].

Lastly, *Lactobacillus* spp., a Gram-positive facultative anaerobe, is widely recognized for its probiotic properties. It aids in SCFA production and exhibits anti-inflammatory activity. It is frequently used in probiotic formulations and is linked to obesity, stress, and autism [97].

Understanding the role of these bacteria in health and disease highlights the importance of maintaining a balanced microbiome. Future research may offer insights into targeted interventions to manipulate microbial populations for improved health outcomes.

One of the main causes of dysbiosis is the use of antibiotics, which destroy both pathogenic and beneficial microbes, as they disrupt the competitive exclusion mechanism through which the microbiota inhibits pathogens [100].

The administration of antibiotics has specific effects on the gut microbiota, depending on the type of antibiotic, the dosage, and the duration of the treatment [101]. For example, the administration of clindamycin for two years causes changes in the gut microbiota without recovery of Bacteroides [102]; the use of clarithromycin in the treatment of *Helicobacter pylori* leads to a decrease in the number of Actinobacteria; ciprofloxacin causes a reduction in *Ruminococcus*, which does not return to its previous level even six months after the end of treatment [103]; vancomycin treatment reduces Bacteroidetes, *Fuminococcus*, and *Faecalibacterium*, while increasing Proteobacteria species [104].

One of the main consequences of dysbiosis is that this imbalance induces the activation of a pro-inflammatory state, leading to an increase in gut permeability. A positive feedback mechanism is involved, as pro-inflammatory cytokines, such as TNF-α, increase epithelial permeability, further exacerbating chronic systemic inflammation. This results in a greater compromise of the gut barrier and the subsequent entry into systemic circulation of metabolites, toxins, and bacteria, which, by losing their cell wall components (lipoteichoic acid and lipopolysaccharide), promote the pro-inflammatory state [105].

### 2.4. Gut Microbiota Dysbiosis and Autoimmune Diseases

Intestinal microbes can produce or even enhance beneficial metabolites or specific immunomodulatory molecules such as polysaccharide A, SCFAs, and retinoic acid through the fermentation of dietary fibers [106,107,108]. The production of SCFAs and trimethylamine can influence a subject’s pathological and health status [109]. SCFAs play a role in protecting against the progression of certain inflammatory diseases. For instance, propionate and butyrate produced by the gut microbiota have been shown to possess anti-inflammatory properties [108]. Butyrate plays a key role in maintaining barrier integrity, as it can cease histone deacetylase activity, causing an increase in regulatory cells that influence wound healing and the differentiation of hair follicle stem cells. Butyrate, primarily produced by *Faecalibacterium prausnitzii*, functions to decrease oxidative stress, provide energy to colonocytes, and activate Treg cells, allowing anti-inflammatory action and conferring immune tolerance to sites beyond the gastrointestinal system [110]. Consequently, a decline in both propionate- and butyrate-producing bacteria can trigger a pro-inflammatory state of the gut and affect intestinal barrier integrity, as well as bacterial translocation beyond its area and immune system stimulation, resulting in a psoriatic phenotype [83]. Moreover, SCFAs are involved in apoptosis and immune cell activation [86].

When an imbalance in the composition and biodiversity of gut microbes occurs, it is referred to as gut dysbiosis, which has been associated with psoriasis and many other comorbidities, such as inflammatory arthritis [86]. Gut dysbiosis negatively impacts skin integrity and function. Some microbes influence intestinal barrier function and skin homeostasis. Gut microbes and their metabolites spread to the skin, showing effects on skin physiology. For example, metabolites such as p-cresol and phenol produced by *Clostridioides difficile* (formerly known as *Clostridium difficile*) are biomarkers of gut dysbiosis that enter the bloodstream and accumulate in the skin, decreasing skin moisture, compromising skin barrier integrity and epidermal differentiation, and affecting keratinization [86,111]. Additionally, gut dysbiosis can affect immune homeostasis, leading to inflammation and autoimmune diseases [87].

Alterations in gut microbiota in psoriasis are similar to those observed in patients with IBD. In these two diseases, *Faecalibacterium prausnitzii*, *Bifidobacterium* spp., *Lactobacillus* spp., *Parabacteroides*, and *Coprobacillus* appear to be underrepresented, whereas the abundance of *Salmonella* sp., *Campylobacter* sp., *Helicobacter* sp., *Escherichia coli*, *Alcaligenes* sp., and *Mycobacterium* sp. is increased [83]. Scientific data indicate that excessive gut colonization by *Candida albicans*, *Malassezia*, and *Staphylococcus aureus* can worsen the psoriatic phenotype. The gut microbiota in psoriasis is characterized by an increase in Actinobacteria and Firmicutes, and the Firmicutes/Bacteroidetes (F/B) ratio represents a model of an altered intestinal epithelial barrier. This leads to the stimulation of regulatory T lymphocytes, carbohydrate transport, and bacterial chemotaxis. On the other hand, *Ruminococcus* and *Megasphaera* are underrepresented in the psoriasis microbiota [83].

Some studies have revealed a greater abundance of species such as Lachnospiraceae and Ruminococcaceae, Collinsella aerofaciens, Dorea formicigenerans, and Ruminococcus gnavus and the underrepresentation of Faecalibacterium prausnitzii and Akkermansia muciniphila in the gut microbiota in psoriasis [107,112]. Faecalibacterium prausnitzii plays an important role in gut homeostasis by producing butyrate, which has antioxidant properties, modulates inflammatory response by inhibiting NF-κB, and provides energy to intestinal epithelial cells (enterocytes) [83].

### 2.5. Gut Microbiota in Psoriatic Arthritis

An alteration in the composition of the intestinal microbiota can determine or at least contribute to the onset of diseases, including PsA. The Firmicutes/Bacteroidetes (F/B) ratio is an important marker of the health status of the intestinal microbiota [113]. Disruption of the F/B ratio can be observed in both psoriatic arthritis and psoriasis [88]. A study found that the F/B ratio is higher in psoriasis patients with enterotype 2 (*Prevotella*-predominant) compared to those with enterotype 1 (*Bacteroides*-predominant) and enterotype 3 (*Ruminococcus*-predominant) [114]. Cho et al. [115] observed that a high F/B ratio in the microbiota of healthy men is associated with higher amounts of trimethylamine-N-oxide (TMAO), which acts as a pro-atherogenic metabolite. A study by Scher et al. [88] compared the intestinal microbiota composition of newly diagnosed, untreated PsA patients with that of psoriasis (PsO) patients and healthy individuals. Regarding the fecal microbiota of PsA patients, an overall decrease in microbial diversity was observed compared to that of healthy subjects. When comparing the fecal microbiota of PsA patients with that of PsO patients at the phylum level, a decrease in Firmicutes, Clostridiales, and Verrucomicrobiales, as well as an increase in Bacteroidetes, was observed in PsA patients compared to PsO patients [112]. At the genus level, a reduction in *Akkermansia*, *Ruminococcus*, and *Pseudobutyrivibrio* was detected in the fecal samples of PsA patients compared to those of PsO patients, with similar dysbiosis patterns previously reported in IBD.

Scher et al. [88] also conducted an analysis to assess the relationship between observed dysbiosis patterns and microbial metabolite concentrations in fecal samples. They found a correlation between the abundance of *Akkermansia* and *Ruminococcus* (both reduced) in the fecal samples of PsA patients and the prevalence of medium-chain fatty acids (MCFA), hexanoate, and heptanoate.

When the levels of these microbial metabolites were compared among the three cohorts, a significant reduction in MCFA was observed in PsA and PsO patients compared to healthy subjects. Finally, researchers measured fecal levels of RANKL and found that only 19% of fecal samples from PsA patients had measurable levels of fecal RANKL compared to 30% of samples from healthy individuals and 75% of samples from PsO patients. Interestingly, this protein, which is overexpressed in the serum and synovium of PsA patients, acts as an osteoclast-activating factor, promoting arthritis development. However, in the intestine, it is responsible for the differentiation of lamina propria cells that interact with antigens in the intestinal lumen [116]. Variations in RANKL concentration may occur due to a specific effect of bacteria typical of psoriasis and PsA patients or may indicate a modulatory effect of this molecule on the development of systemic inflammation characteristic of psoriasis.

In summary, the results of Scher et al. [88] indicate a microbial community and specific intestinal metabolites in PsA patients, characterized by reduced fecal MCFA and RANKL levels, which are significantly different not only from healthy individuals but also from PsO patients. A 2019 study by Sharma and colleagues [117] also examined evidence of intestinal inflammation in individuals with PsA compared to those with PsO and irritable bowel syndrome (IBS) using a fecal calprotectin test, a useful marker of intestinal inflammation. Elevated calprotectin levels were observed in 58% of PsA patients, 26% of PsO patients, and 10% of IBS patients. Moreover, they found that the average amount of calprotectin was significantly higher in PsA patients than in PsO patients, and in PsA fecal samples, calprotectin concentration correlated positively with body surface area. These results led to the hypothesis that gastrointestinal inflammation is involved in the onset of PsA or in the transition from PsO to PsA, as calprotectin levels were higher in PsA patients than in PsO patients. It was also inferred that gastrointestinal inflammation may be considered a risk factor for PsA in PsO patients. Another potential effect of the gut microbiota on the disease is microbial translocation, linked to the presence of bacterial DNA in the peripheral blood of patients with PsA, ankylosing spondylitis (AS), and rheumatoid arthritis (RA), in the synovial fluid of patients with chronic arthritis, and in PsA patients [118]. The presence of DNA in the synovial fluid may result from increased permeability of intestinal epithelial barriers. Outer membrane vesicles (OMVs) of gut bacteria are likely vectors of bacterial DNA in the synovium, as they are significantly smaller than bacteria and thus allow the transfer of bacterial nucleic acid into the bloodstream. OMVs play an important role in intestinal homeostasis, as they interact with the immune system and can activate dendritic cells (DCs) through TLR2, whose expression is upregulated in DCs of PsA patients [119].

The genera *Prevotella*, *Akkermansia*, *Faecalibacterium*, and *Ruminococcus* have been observed in reduced quantities in psoriasis [120,121] and PsA [88]. As suggested by several studies, they are all symbionts that produce SCFAs, promoting intestinal health. A similar underrepresentation with reduced *Akkermansia, Ruminococcus*, and *Faecalibacterium* was also observed in studies on the gut microbiome of IBD patients [72]. Additionally, *Akkermansia* reduction is associated with obesity, metabolic syndrome, and aging [122,123], while *Faecalibacterium* reduction has been observed in atopic dermatitis [124].

Overall, these findings suggest that intestinal dysbiosis in psoriasis and PsA is characterized by a loss of butyrate-producing bacteria. The reduction in these bacteria plays a role in the pathogenesis of psoriasis and PsA, allowing a local inflammatory response that, in turn, impairs the function of the intestinal epithelial barrier and its role in regulating the presentation of gastrointestinal antigens to immune cells and systemic circulation [125]. Furthermore, *Faecalibacterium* spp. and *Akkermansia* spp. have been shown to suppress Th17 and induce the development and expansion of regulatory T cells (Tregs), a cell type integral to maintaining immune tolerance, producing anti-inflammatory cytokines, and preventing autoimmunity [126,127]. Eppinga et al. [128] suggested that *Faecalibacterium* spp. and possibly *Akkermansia* spp. can negatively alter the immune system both in the gut and systemically when present in insufficient amounts. These insights, besides aiding in understanding the disease and its pathogenesis, are important as they may have therapeutic implications.

## 3. Future Perspectives: The Microbiota as a Therapeutic Target

Numerous therapeutic strategies have been developed to rebalance the intestinal ecosystem and treat diseases: probiotics, prebiotics, fecal transplantation, and phage therapy [129].

### 3.1. Probiotics

Probiotics are live microorganisms generally considered safe (GRAS) that, when taken in adequate quantities, confer health benefits. They are active and viable in the intestinal environment and resistant to bile and pancreatic secretions of the gastrointestinal environment [130,131]. The functions of probiotic bacteria are multiple and variable: they lower intestinal pH by producing SCFA, synthesize vitamins such as B and K, metabolize carcinogenic substances, and exhibit antimicrobial activity against pathogenic microbes through the production of bacteriocins and other inhibitory substances. Additionally, they stimulate the immune response, both directly by increasing macrophage activity and modulating the secretion of immunoglobulins and cytokines, and indirectly by strengthening the intestinal epithelial barrier and altering mucus secretion [130,132].

The most commonly used probiotic species include bacteria belonging to the genera *Lactobacillus* and *Bifidobacteria*, as well as yeasts such as *Saccharomyces boulardii* [133]. Depending on the clinical context, probiotics can be administered as drugs or combined with foods such as yogurt and dairy products. Probiotics are widely marketed as supplementary and functional foods, including yogurt, cheese, chocolate, ice cream, and non-dairy food products [134]. Many probiotic industries use microencapsulation techniques to protect bacteria from environmental factors.

Probiotics can also be used to prevent the onset of dysbiosis, which occurs when patients are exposed to certain conditions such as prolonged antibiotic therapy, intense physical or mental stress, and chronic diseases. They can also be used as therapeutic agents to rebalance ongoing dysbiosis. The benefits of probiotics depend on the dose and duration of administration, strain selection, and gastrointestinal tract retention [135].

Probiotics also prevent cardiovascular diseases by regulating lipid metabolism, specifically by reducing cholesterol absorption through co-precipitation with deconjugated bile salts, incorporating and assimilating cholesterol into the cell membrane, converting cholesterol into coprostanol, and inhibiting cholesterol transporter expression in enterocytes [136]. Probiotics have been reported as a therapeutic alternative for reducing various diseases, as shown in Table 4.

Although there are no available data on the effect of probiotics in patients with PsA, some studies have demonstrated that probiotic use has a positive effect on the course of psoriasis [149,150]. The species *Lactobacillus pentosus* GMNL-77 and *Bifidobacterium infantis* 35,624 improved psoriasis in murine models (with psoriasis induced by Imiquimod, a topical drug) and led to a decrease in plasma CRP values and pro-inflammatory cytokines (TNF-α and IL-6). In humans, this indicates a potential anti-inflammatory role of probiotics [151,152]. The influence of probiotics on the immune system likely occurs due to the down-regulation of dendritic cells, which play a role in antigen presentation to regulatory T cells in the human intestine [153].

### 3.2. Prebiotics

Prebiotics are described as selectively fermented ingredients that cause specific changes in the organization and functions of the gastrointestinal microbiota, conferring benefits to the host [154]. The European Union has approved prebiotics (inulin, FOS, and GOS) as safe food ingredients, while the FDA has not yet recognized them.

In the gastrointestinal tract, prebiotics resist gastric acidity and reach the colon, where they are processed by the microbiota, leading to SCFA production [155]. Prebiotics such as FOS and GOS also modulate neural growth factors, such as brain-derived neurotrophic factor, neurotransmitters, and synaptic proteins, including synaptophysin [156]. Therefore, it can be stated that the utilization of prebiotics by the intestinal microbiota can confer health benefits across different body regions [155]. Experimental evidence has also suggested that prebiotic supplementation is effective against impaired memory, Alzheimer’s disease, and dementia [157].

### 3.3. Fecal Microbiota Transplantation

Fecal microbiota transplantation (FMT) is the process of transplanting fecal microorganisms from healthy individuals to patients with specific diseases. There is substantial evidence supporting FMT as a highly effective therapeutic option for various intestinal diseases, as it restores the composition and functions of the recipient′s intestinal microbiota [158].

FMT has been widely used to treat many diseases, including irritable bowel syndrome, inflammatory bowel disease, insulin resistance, obesity, autism, diarrhea, allergic disorders, metabolic syndrome, colon cancer, anti-tumor immunity, neuropsychiatric diseases, and Parkinson’s disease [159,160].

The exact mechanism by which FMT acts in many diseases is still unknown; it could be due to changes in bacterial composition, alterations in the host’s metabolic profiles, or the involvement of new microbiota species present in the donor’s healthy stool. Despite its many advantages, FMT may also have side effects, such as the risk of transferring microbial pathogens or creating safety concerns due to the complexity of the fecal microbial community [158].

To overcome these disadvantages, researchers are considering using defined fecal microbiota preparations composed only of therapeutic microorganisms, or using mixtures of defined species or strains, or cocktails of microbiota-derived molecules targeted at specific pathological states [161].

### 3.4. Phage Therapy

Among future perspectives for treating diseases by modifying the intestinal microbiota composition is phage therapy.

Phages constitute about 90% of the human virome and have a significant influence on bacterial populations. Therefore, they have great therapeutic potential and could be used both for antimicrobial purposes and to modulate microbial community composition [162]. An important feature of phages is that they multiply exponentially after administration, although their growth kinetics are not constant but depend on the concentration of susceptible bacteria and the human host’s immune responses. These factors make it difficult to determine the exact dosage and administration timing [163]. Consequently, further studies are needed before phages can be approved as drugs.

These therapeutic approaches hold promise for managing PsA and other microbiota-related diseases, paving the way for personalized medicine interventions.

### 3.5. Diet

Emerging evidence suggests that dietary patterns play a crucial role in shaping gut microbiota composition, which in turn may influence inflammation and disease progression in PsA. The Mediterranean diet, rich in fiber, polyphenols, and omega-3 fatty acids, has been associated with increased microbial diversity and reduced systemic inflammation, potentially benefiting PsA patients [164,165]. Specific dietary components such as polyphenols from fruits, vegetables, and olive oil have shown prebiotic-like effects, promoting beneficial gut bacteria and reducing pro-inflammatory species [166]. Likewise, omega-3 fatty acids from fish and nuts have been linked to shifts in microbiota composition that favor anti-inflammatory pathways [167]. These dietary strategies may represent adjunctive therapeutic approaches to complement conventional PsA treatments by targeting the gut-joint axis.

### 3.6. Challenges in Microbiota-Based Therapy

While probiotics and FMT have been proposed as promising therapeutic strategies for modulating gut microbiota in PsA, their clinical effectiveness and safety remain areas of ongoing investigation. Probiotic supplementation has shown potential in improving gut dysbiosis and systemic inflammation in inflammatory arthritis models, yet clinical trials in PsA are still limited and heterogeneous in design [168,169]. Additionally, the efficacy of probiotics may vary depending on strain selection, dosage, and patient-specific microbiota composition, raising concerns about standardized treatment protocols. Similarly, FMT has emerged as a novel approach to restoring microbial balance; however, its application in PsA lacks robust clinical evidence. While early trials in IBD, which shares pathophysiological similarities with PsA, suggest benefits in modulating immune responses its direct efficacy in PsA remains largely theoretical. Moreover, safety concerns, including the risk of pathogen transmission, unintended immune activation, and variability in donor microbiota composition, necessitate further investigation. Given these limitations, microbiota-based interventions should be approached cautiously, emphasizing the need for well-designed clinical trials to establish their therapeutic potential in PsA management.

## 4. Conclusions

Growing evidence suggests that the gut microbiota plays a significant role in the PsA pathogenesis by influencing immune system function and systemic inflammation. Dysbiosis, or an imbalance in microbial composition, has been associated with chronic inflammatory diseases, including PsA, potentially contributing to disease onset and progression. The interaction between gut microbiota and immune pathways may impact key inflammatory mediators, such as TNF-α, IL-6, and IL-17, which are central to PsA pathophysiology.

Modulating the microbiota through probiotics, prebiotics, FMT, and phage therapy may offer new therapeutic options for PsA. While specific data on the effects of probiotics in PsA patients are lacking, studies on psoriasis suggest potential benefits in immune regulation and systemic inflammation reduction. Prebiotics, by promoting SCFAs production and influencing neural and immune pathways, may provide additional health benefits. FMT has shown efficacy in treating several gastrointestinal and metabolic disorders, with growing interest in its application for autoimmune and neuroinflammatory conditions. However, concerns about safety and pathogen transmission highlight the need for refined approaches, such as defined microbiota preparations. Phage therapy, a promising yet experimental strategy, could be used to selectively modulate microbial communities, though further research is required to establish clinical applications.

Beyond potential therapeutic benefits, the gut microbiota may also serve as a biomarker to guide the selection of biologic drugs and small-molecule therapies for PsA. The composition and metabolic activity of gut microbiota could influence individual responses to targeted therapies, such as TNF inhibitors, IL-17 and IL-23 blockers, JAK inhibitors. A deeper understanding of microbiota-related mechanisms may pave the way for precision medicine approaches, enabling more personalized and effective treatment strategies for PsA patients.

Overall, targeting the gut microbiota represents an exciting frontier in PsA research, offering potential insights into disease mechanisms, therapeutic interventions, and personalized treatment selection. Further studies and clinical trials are essential to validate these approaches and translate them into routine clinical practice.

## Figures and Tables

**Table 1:** Potential risk factors for the development of PsA.

Category	Risk Factor	Details
Non-modifiable Factors	Family history of PsA	Genetic predisposition
	Genetics	Inherited risk
	Psoriasis-related factors	-Age of psoriasis onset -Severity -Localization -Nail involvement
Modifiable Factors	Environmental factors	-Physical trauma -Bacterial infections
		-Vaccination
	Metabolic abnormalities	-Obesity -Hyperlipidemia -Hyperuricemia
	Smoking	Increased inflammation risk
	Medications	NSAIDs (non-steroidal anti-inflammatory drugs)

**Table 2:** Main cytokines involved in PsA pathogenesis.

Cytokine	Expression Site	Source	Key Functions
TNF-α	Synovial tissue, synovial fluid	Macrophages, T cells, fibroblast-like synoviocytes, B cells	Activates immune cells, induces cytokine and matrix metalloproteinase (MMP) production, promotes cartilage resorption.
Interleukin-23 (IL-23)	Synovial tissue, synovial fluid, enthesis	Macrophages, dendritic cells	Promotes Th17 cell differentiation and production of granulocyte-macrophage colony-stimulating factors (GM-CSF).
Interleukin-17A/F (IL-17)	Synovial tissue, synovial fluid, enthesis	T cells, mast cells, natural killer cells	Stimulates fibroblast-like synoviocytes, chondrocytes, osteoclasts, and production of inflammatory cytokines and MMPs; recruits neutrophils.
Interleukin-22 (IL-22)	Synovial tissue, synovial fluid, enthesis	T cells, innate lymphoid cells	Activates fibroblast-like synoviocytes, induces osteoclastogenesis, and promotes bone resorption through RANKL expression.

**Table 3:** Overview of bacterial species and their impact on human health.

Bacterial Species	Basic Features	Associated Physiological Changes	Associated Disease States	References
*Akkermansia muciniphila*	Gram-negative obligate anaerobe	Anti-inflammatory effects	↓ in IBD, obesity, and T2D (↑ after metformin treatment); ↑ in fish-oil-fed mice; ↓ after cold exposure	[96,97]
*Bacteroides* spp.	Gram-negative obligate anaerobe	Activate CD4+ T cells	↑ with animal-based diet and obesity; *Bacteroides vulgatus* positively correlates with IR	[90]
*Bifidobacterium* spp.	Gram-positive obligate anaerobe	SCFA production; improve gut mucosal barrier; lower intestinal LPS levels	↓ in obesity and smokers; ↑ in RTT syndrome; used as probiotic	[98,99]
*Bilophila* spp.	Gram-negative obligate anaerobe	Promote pro-inflammatory immunity	↑ in colitis; ↑ in lard-fed mice; ↓ in autism	[96]
*Christensenella* spp.	Gram-negative anaerobe	Negative correlation with BMI	*Christensenella minuta* decreased weight gain after transplant	[97]
*Clostridium* spp.	Gram-positive obligate anaerobe	Promote generation TH17 cells	Several spp. cause botulism, tetanus, and other diseases; ↑ after sidestream smoke exposure; ↓ in IBD; ↑ in autism, RTT syndrome, T2D	[96,97]
*Dialister* spp.	Gram-positive obligate anaerobe	Some spp. are pathogenic	↑ in obesity and periodontitis; ↓ in autism	[96]
*Enterobacter* spp.	Gram-negative facultative anaerobe	Several spp. are pathogenic	↓ after side-stream smoke exposure; *Enterobacter cloacae* induces obesity in germ-free mice	[90]
*Escherichia coli*	Gram-negative facultative anaerobe	TLR activation	↑ in IBD and T2D	[98]
*Eubacterium* spp.	Gram-positive obligate anaerobe	SCFA and phenolic acids production	↓ in IBD, atherosclerosis, and T2D; *Eubacterium saphenum* ↑ in periodontitis	[99]
*Faecalibacterium prausnitzii*	Gram-positive obligate anaerobe	SCFA production and anti-inflammatory effects	↓ in IBD, obesity, T2D, and overweight	[96]
*Lactobacillus* spp.	Gram-positive facultative anaerobe	SCFA production; anti-inflammatory activity	Attenuate IBD; ↑ in fish oil-fed mice; used as probiotic; linked to obesity, stress, and autism	[97]
*Roseburia* spp.	Gram-positive obligate anaerobe	SCFA production	↓ in IBD, obesity, T2D, and atherosclerosis	[96]
*Streptococcus* spp.	Gram-positive facultative anaerobe	Some spp. are pathogenic	*S. mutans* ↑ in oral cavity after high-carb diet, linked to caries; *S. salivarius* used as probiotic for periodontitis	[97]
*Veillonella* spp.	Gram-negative obligate anaerobe	Fermentation of lactate to propionate and acetate	↑ in oral cavity after smoking; ↓ in autism	[96]
*Prevotella* spp.	Gram-negative obligate anaerobe	Some spp. cause infections in oral and respiratory tract	↑ with high-fiber diet; *P. copri* linked to insulin resistance; ↓ in autism and Parkinson’s disease	[98]
*Porphyromonas* spp.	Gram-negative obligate anaerobe	Some spp. are pathogenic	*P. gingivalis* and *P. endodontalis* linked to periodontitis; ↓ in smokers; ↑ in obesity	[96]
*Neisseria* spp.	Gram-negative obligate aerobe	Sugar fermentation	Only *N. meningitidis* and *N. gonorrhoeae* are pathogenic; ↓ in smokers	[97]

BMI: body mass index; IBD: inflammatory bowel disease; IR: insulin resistance; RTT: Rett syndrome; SCFA: short-chain fatty acids; T2D: type 2 diabetes; TLR: Toll-like receptor. ↑: increase; ↓: reduction.

**Table 4:** Some therapeutic strategies used to rebalance the gut microbial ecosystem.

Therapeutic Strategy	Disease	Achieved Results	References
*Akkermansia muciniphila*	-Obesity -Metabolism disorders -Diabetic subjects	-Re-equilibration of gut microbiota dysbiosis -Reversion of atherosclerotic lesions	[137,138]
*Faecalibacterium prausnitzii*	-Gut microbiota dysbiosis	-Re-equilibration of gut microbiota dysbiosis -Protective effects -Production of short fatty acids (SCFAs)	[139,140]
*Bacteroides uniformis*	-High fat diet -Ulcerative colitis	-Enhancement of lipid profiles, leptine and glucose level -Reduction in colon contraction -Improvement of gut bleending -Attenuation of mucosal damage	[141,142]
*Bdellovibrio bacteriovorus*: a specialized bacterial predator	-Gram-negativeGram-negative infections -Gut microbiota dysbiosis (with overgrowth of Gram negative bacteria)	Re-equilibration of gut microbiota dysbiosis	[143,144]
Bacteriotherapy	-Gut microbiota dysbiosis -*Clostridioides difficile* infections -Ulcerative colitis	Re-equilibration of gut microbiota dysbiosis	[145,146]
Phage therapy	-Bacterial infections -Gut microbiota dysbiosis	Re-equilibration of gut microbiota dysbiosis	[147,148]
